# Mitochondrial Interactome: A Focus on Antiviral Signaling Pathways

**DOI:** 10.3389/fcell.2020.00008

**Published:** 2020-02-14

**Authors:** Giulia Refolo, Tiziana Vescovo, Mauro Piacentini, Gian Maria Fimia, Fabiola Ciccosanti

**Affiliations:** ^1^Lazzaro Spallanzani, National Institute for Infectious Diseases – IRCCS, Rome, Italy; ^2^Department of Biology, University of Rome Tor Vergata, Rome, Italy; ^3^Department of Molecular Medicine, Sapienza University of Rome, Rome, Italy

**Keywords:** mitochondrial antiviral signaling protein, retinoic acid-inducible gene I, proteomics, mitochondria, RNA virus infection

## Abstract

In the last years, proteomics has represented a valuable approach to elucidate key aspects in the regulation of type I/III interferons (IFNs) and autophagy, two main processes involved in the response to viral infection, to unveil the molecular strategies that viruses have evolved to counteract these processes. Besides their main metabolic roles, mitochondria are well recognized as pivotal organelles in controlling signaling pathways essential to restrain viral infections. In particular, a major role in antiviral defense is played by mitochondrial antiviral signaling (MAVS) protein, an adaptor protein that coordinates the activation of IFN inducing pathways and autophagy at the mitochondrial level. Here, we provide an overview of how mass spectrometry-based studies of protein–protein interactions and post-translational modifications (PTMs) have fostered our understanding of the molecular mechanisms that control the mitochondria-mediated antiviral immunity.

## Introduction

Mitochondria are the powerhouse of the cell due to their primary contribution in cell respiration. In addition to their canonical role in cellular metabolism, mitochondria have emerged as a central platform for the regulation of antiviral signaling pathways (Mohanty et al., 2019). Although several pathogen-activated pathways are influenced by mitochondria, this role is to be attributed mainly to the mitochondrial localization of the signaling adaptor mitochondrial antiviral signaling (MAVS) protein, a key mediator of the innate immune response during RNA viral infection (Vazquez and Horner, 2015).

In the last years, remarkable advances have been made in our knowledge about the molecular mechanisms underlying MAVS signaling. The development of innovative technologies in the field of transcriptomics, proteomics, reverse genetics, and structural and cell biology has dramatically improved our understanding of how RNA viruses can be sensed and lead to the expression of hundreds of genes dedicated to infection inhibition. In particular, yeast two-hybrid and mass spectrometry (MS) approaches have provided detailed information on how MAVS-dependent antiviral responses are rapidly activated, and also tightly controlled by protein catabolic processes, i.e. the ubiquitin–proteasome system and autophagy, to avoid excessive inflammation and cell death. Moreover, these approaches have widened our understanding of how viral proteins are able to inhibit MAVS activation to accomplish viral replication and how energy metabolism may influence mitochondrial antiviral pathways.

Here, we review our current view of MAVS signaling, highlighting how proteomics has deepened our understanding of this pathway regulation in terms of protein–protein interactions and post-translational modifications (PTMs).

## Innate Immunity and Mitochondria

The innate immune response is the first line of defense against invading pathogens that is activated following the recognition of specific entities, called pathogen-associated molecular patterns (PAMPs), through a series of receptors, termed pattern recognition receptors (PRRs). Upon detection of PAMPs, the transcription of a myriad of antiviral genes is activated, establishing a cellular antiviral state that helps cells to restrict and/or clear infection (Brubaker et al., 2015).

Based on their structures, locations, and functional specificities, PRRs are separated into discrete families, which include the membrane bound Toll-like receptors (TLRs), the cytosolic nucleotide-binding oligomerization domain (NOD)-like receptors (NLRs), cyclic GMP-AMP (cGAMP) synthase (cGAS), and retinoic acid-inducible gene I (RIG-I)-like receptors (RLRs) (Kawai and Akira, 2011; Franchi et al., 2012; Streicher and Jouvenet, 2019). The activity of these PRRs has been described to be influenced at various levels by mitochondria.

The TLRs are a family of transmembrane PRRs that are activated by the binding of ligands to their C-terminal leucine-rich repeats. Ten TLRs have been identified in humans, with TLRs 1, 2, 4, 5, 6, and 10 located at the cell surface and TRLs 3, 7, 8, and 9 spanning the endosomal membrane. Plasma membrane TLRs mainly recognize microbial wall components, such as lipids, polysaccharides, and proteins, while intracellular TLRs sense pathogen nucleic acids (Kawai and Akira, 2011). Stimulation of TLRs results in the activation of the transcription factors NF-κB and IRF3/IRF7 to induce the expression of type I interferon (IFN) genes and other inflammatory cytokines.

Mitochondria are directly involved in the regulation of TLR activity. A relevant example is represented by TLR1, TLR2, and TLR4, which were reported to enhance mitochondrial ROS generation in bacteria-infected macrophages to potentiate the antimicrobial killing (West et al., 2011). This effect is mediated by evolutionarily conserved signaling intermediate in Toll (ECSIT) pathways, a mitochondrial matrix protein required for mitochondrial respiratory complex I activity. Following TLR engagement, the E3 ubiquitin ligase tumor necrosis factor receptor-associated factor 6 (TRAF6) binds to a fraction of the ECSIT protein that becomes accessible on the mitochondrial surface and elicits the pro-oxidant activity of ECSIT via non-degradative ubiquitination (West et al., 2011). Moreover, TLR4 activation drives the formation of a complex including TRAF6, ECSIT, and transforming growth factor-β-activated kinase 1 (TAK1), which promotes TAK1 kinase activity and the activation of the NF-κB signaling (Wi et al., 2014).

The NLR family of PRRs are cytoplasmic proteins that stimulate innate immunity in response to both PAMPs, such as microbial components (e.g. peptidoglycan, viral RNA), and damage-associated molecular patterns (DAMPs), including host components (cholesterol crystals, uric acid), and environmental sources (alum, asbestos, skin irritants) (Franchi et al., 2012). Upon activation, a unique feature of NLRs is the formation of a multiprotein complex termed “inflammasome.” Here, the NLRs bind pro-caspase-1 through the adaptor apoptosis-associated speck-like protein containing a caspase activation and recruitment domain (CARD) (ASC). Inflammasome assembly triggers auto-catalysis of pro-caspase-1, which is able to process the pro-IL (interleukin)-1β and pro-IL-18 into their active forms in order to induce, once secreted, a broad inflammatory response (Sharma and Kanneganti, 2016).

A direct link between mitochondria and NLR activation has been reported for NLRP3. Active NLRP3 translocates from cytosol to mitochondria, where it functions as a scaffold for inflammasome assembly (Swanson et al., 2019). Here, NLRP3 was found to interact with MAVS, with this binding being necessary for optimal inflammasome activity (Subramanian et al., 2013).

Cyclic GMP-AMP synthase is one of the cellular sensors of non-self DNA, together with the endosomal TLR9 and the NLR family protein Absent in Melanoma 2 (AIM2). Cytosolic DNA binds to cGAS and stimulates its enzymatic activity to form the second messenger cGAMP (Wu et al., 2013). cGAMP binds to the endoplasmic reticulum (ER) resident protein STING, promoting a conformational change that allows STING to interact with the protein kinases TBK1 (TANK binding kinase-1) and IKKs (IκB kinases) and to translocate from the ER to the Golgi apparatus. Activated TBK1 and IKKs phosphorylate the transcription factors IRF3 and NF-κB so as to induce the expression of type I IFN and inflammatory cytokines (Motwani et al., 2019).

Although DNA and RNA sensing responses occur through independent receptors, the induction of the antiviral response by one of these agents also relies on the activity of the other pathway. Multiple molecular mechanisms are responsible for this strict interdependence, including the direct interaction of the main regulators of these pathways at the ER–mitochondria contact sites, as well as the fact that many downstream factors are shared between these pathways. For a more exhaustive review of the crosstalk between RNA and DNA sensing pathways, see Zevini et al. (2017).

## The RIG-I-Like Receptor Signaling Pathways

**FIGURE 1:**
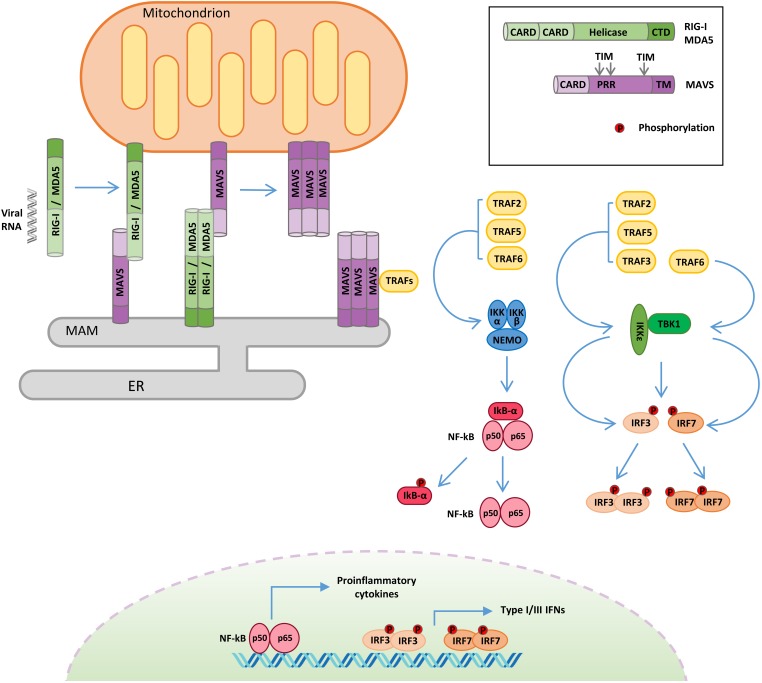
Retinoic acid-inducible gene I-like receptor (RLR)–mitochondrial antiviral signaling (MAVS) protein signaling. Following viral RNA sensing, retinoic acid-inducible gene I (RIG-I)/melanoma differentiation associated gene 5 (MDA5) oligomerize and relocalize from the cytoplasm to outer mitochondrial membrane (OMM) and mitochondria-associated membrane (MAM). RIG-I/MDA5 interaction with MAVS induces MAVS oligomerization and the formation of a signalosome where the tumor necrosis factor receptor-associated factor (TRAF) proteins are recruited. TRAF proteins trigger two molecular cascades leading to: (i) IRF3 and IRF7 phosphorylation by TANK binding kinase-1 (TBK1) and IκB kinase ε (IKKε) to induce the expression of type I and type III interferons (IFNs) and (ii) NF-κB phosphorylation by the IKK α/β/γ complex to upregulate the expression of inflammatory cytokines. TIM: TRAF-interacting motif. PRR: proline-rich region. ER: endoplasmic reticulum.

Apart from the endosome localized TLRs, the recognition of non-self RNA is mediated by the cytosolic sensors of the RLR pathway (Figure 1).

The RLR family contains three members: RIG-I, melanoma differentiation associated gene 5 (MDA5), and laboratory of genetics and physiology 2 (LGP2). All RLRs are characterized by a central DEAD box helicase/ATPase domain and a C-terminal regulatory domain (CTD), necessary for RNA binding and to prevent constitutive activation (Tan et al., 2018).

Moreover, RIG-I and MDA5 possess two N-terminal CARDs that mediate their oligomerization following RNA binding and are required for the activation of the downstream adaptor protein MAVS (Brisse and Ly, 2019). RIG-I and MDA5 play non-redundant roles by detecting largely distinct groups of viruses and by recognizing distinct features of viral RNAs. RIG-I preferentially detects 5′-di-/triphosphorylated RNA sequences rich in poly-U or poly-UC tracts, whereas MDA5 binds to high-molecular-weight viral RNAs (Kato et al., 2006). LGP2 exhibits 30–40% amino acid sequence identity to RIG-I and MDA5, but it lacks the CARDs (Yoneyama et al., 2005). The exact role of LGP2 in innate immunity remains not completely understood, since it has been reported to act both as a positive (Venkataraman et al., 2007; Satoh et al., 2010) and as a negative (Komuro and Horvath, 2006; Parisien et al., 2018) regulator of the RLR pathway. To explain these discrepancies, it has been recently proposed that LGP2 may function as a concentration-dependent switch. Early during infection, low levels of LGP2 enhance RLR-mediated antiviral signaling, while, at later stages, IFNs stimulate the expression of LGP2, which, at high concentration, inhibits RLRs, contributing to the termination of the antiviral response (Bruns and Horvath, 2015).

The adaptor protein MAVS (also known as IPS-1/VISA/Cardif) is essential to drive innate immunity in response to RNA virus infection (Figure 1). MAVS is comprised of three functional domains, a CARD at the N terminus, a proline-rich domain, and a C-terminal membrane-targeting transmembrane domain. MAVS contains three TRAF-interacting motifs (TIMs), two in the proline-rich region and one near the transmembrane domain (Seth et al., 2005; Vazquez and Horner, 2015). Through its C-terminal transmembrane domain, MAVS is anchored to different subcellular districts, including the outer mitochondrial membrane (OMM), peroxisomes, and a subdomain of the ER called the mitochondria-associated membrane (MAM) (Seth et al., 2005; Dixit et al., 2010; Horner et al., 2011). It has been proposed that the differential localization of MAVS may drive distinct antiviral signaling responses, with the mitochondrial protein promoting type I IFN expression, while the peroxisomal protein signals for the induction of the type III IFN (Odendall et al., 2014). Other evidence supports the involvement of peroxisomal MAVS in the early induction of IFN-stimulated genes (ISGs), before mitochondrial MAVS establishes a sustained antiviral response (Dixit et al., 2010). Anyhow, these organelles are tightly connected, and mitochondria and peroxisomes interact with each other in signaling “synapses” during activation of the RIG-I pathway (Horner et al., 2011). Moreover, the different localization could also be related to the ability of MAVS to regulate other processes, as in the case of MAM, where MAVS can interact with regulators of cellular metabolism and apoptotic program (Hayashi et al., 2009).

Mitochondrial antiviral signaling-dependent antiviral signaling initiates after RIG-I and MDA5 sense viral RNA in infected cells (Figure 1). In the absence of viral RNA, the CARDs of RIG-I/MDA5 are masked by intramolecular interactions with the helicase domain, but upon viral RNA binding, an ATP-mediated conformational change allows the formation of oligomers, which properly expose their CARDs so as to interact with the equivalent domain of MAVS (Kowalinski et al., 2011). Moreover, RIG-I oligomers form a complex with 14-3-3ε, a mitochondria-targeting chaperone that mediates their translocation from the cytoplasm to OMM and MAM, where MAVS is localized (Liu et al., 2012). The association of RIG-I/MDA5 with MAVS triggers the formation of detergent-insoluble aggregates on the surface of mitochondria (Tang and Wang, 2009). This conformation is the active state of MAVS, necessary for recruiting the downstream effectors TRAF3, TRAF6, TRAF2, and TRAF5 in order to form a signaling supramolecular complex defined as the “MAVS signalosome” (Figure 1) (Hou et al., 2011).

Interestingly, the spontaneous aggregation of MAVS in the absence of viral RNA is prevented by multiple N-terminal truncated proteins that are encoded by the MAVS transcript (Qi et al., 2017). In addition, the inhibition of the short MAVS proteins also results in the autophagic degradation of the full-length protein, highlighting a role of these isoforms in the control of MAVS stability in resting conditions (Brubaker et al., 2014).

Once recruited to the MAVS signalosome, TRAF proteins stimulate two molecular cascades leading to: (a) the phosphorylation of the transcription factors IRF3 and IRF7 by TBK1 and IKKε to induce the expression of type I/III IFNs and (b) the phosphorylation of the transcription factor NF-κB by the IKKα/β/γ complex to upregulate the expression of inflammatory genes (Liu et al., 2013). In turn, type I/III IFNs induce the expression of hundreds of ISGs to establish a protective state, eventually providing viral clearance of the infected cells, while inflammatory cytokines are mainly responsible for regulating the activity of innate and adaptive immune cells (Lazear et al., 2019; Tait Wojno et al., 2019).

## Regulation of Mavs Signaling by Post-Translational Modifications

**FIGURE 2:**
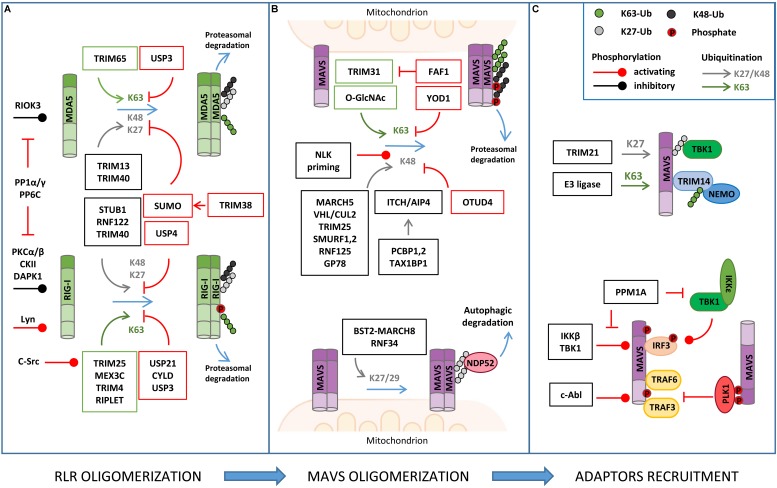
Regulation of RLR–MAVS signaling by post-translational modification (PTM). **(A)** RLR oligomerization is regulated by different E3 ligases that catalyze lysine 63 (K63)-linked ubiquitination (K63) of both RIG-I and MDA5 to promote oligomerization. K63-linked ubiquitination is countered by different DUBs (USPs, CYLD). K48- or K27-linked ubiquitination directs RIG-I/MDA5 to proteasomal degradation. USP and sumoylation inhibit K48-linked ubiquitination and RLR degradation. Phosphorylation levels regulated by the indicated kinases and phosphatases also regulate RIG/MDA5 oligomerization. **(B)** MAVS activity is tightly regulated by K63-linked ubiquitination of MAVS, promoting its oligomerization, and K48-linked ubiquitination, which triggers protein degradation. K48-linked ubiquitination is primed by NEMO-like kinase (NLK) and countered by ovarian tumor family deubiquitinase 4 (OTUD4) (upper panel); K27/K29-linked ubiquitination mediates NDP52-dependent autophagic degradation of MAVS (lower panel). **(C)** MAVS interaction with downstream effectors is regulated by non-degradative ubiquitination (K27- or K63-linked) and phosphorylation, as indicated.

Mass spectrometry-based analyses combined to site-specific mutagenesis approaches have greatly contributed to understand how PTM controls MAVS antiviral response (Figure 2). This has been possible thanks to the improvement of methodologies for PTM enrichment and liquid chromatography-tandem MS (LC-MS/MS)-mediated detection (Yates, 2019). These proteomic studies allowed the identification of hundreds of modification sites in MAVS signaling proteins, whose role in the antiviral response has been extensively investigated, as described hereafter in detail.

The main PTM responsible for the regulation of RIG-I, MDA5, and MAVS activity is ubiquitination, which acts as either a degradative or regulatory signal, depending on the type of polyubiquitin chain that is formed (Figure 2 and Table 1).

**TABLE 1:** MAVS post-translational modifications.

Modified amino acids	Methods	Effects of modification on MAVS	References
***Phosphorylation***			
S328/S330	MS/MS and mutagenesis	Activation of IRF3/7 and NF-κB.	Kandasamy et al., 2016
Y9	Mutagenesis	Recruitment of TRAF3 and TRAF6 to MAVS	Song et al., 2010
Y11/Y30/Y71	Mutagenesis	Inhibition of MAVS–LC3 interaction to prevent autophagy-mediated MAVS degradation	Cheng et al., 2017
T234/S233	Mutagenesis	Recruitment of PLK1 to inhibit MAVS activation	Vitour et al., 2009
S442	MS/MS and mutagenesis	Mediated by TBK1 and IKK to recruit IRF3 binding and activation	Liu et al., 2015
S121/S212/S258/S329	MS/MS and mutagenesis	Phosphorylation of MAVS by NLK causes its degradation	Li et al., 2019
***Ubiquitination***			
K7/K500	Mutagenesis	K48-linked ubiquitination by MARCH5 to promote MAVS proteasomal degradation	Yoo et al., 2015
K7/K10	MS/MS and mutagenesis	K48-linked ubiquitination by TRIM25 to promote MAVS proteasomal degradation	Castanier et al., 2012
K7	Mutagenesis	K27-linked ubiquitination by MARCH8 for NDP52-dependent autophagic degradation of MAVS	Jin et al., 2017
K362/K461	Mutagenesis	K48-linked ubiquitination by RNF5 for MAVS proteasomal degradation	Zhong et al., 2010
K10/K311/K461	Mutagenesis	K63-linked polyubiquitination by TRIM31 to promote the aggregation and activation of MAVS	Liu et al., 2017
K371/K420	Mutagenesis	K48-linked ubiquitination by AIP4 for MAVS proteasomal degradation	You et al., 2009
K420	Mutagenesis	K48-linked ubiquitination by pVHL for proteasomal degradation of MAVS	Du et al., 2015
K371/K420/K500	MS/MS and mutagenesis	K11-linked ubiquitination by TRIM29 for proteasomal degradation of MAVS	Xing et al., 2018
K325	Mutagenesis	K27-linked polyubiquitination by TRIM21 to promote association with TBK1	Xue et al., 2018
K297/K311/K348 and K362	MS/MS and mutagenesis	K27-/K29-linked polyubiquitination by RNF34 for NDP52-dependent autophagic degradation	He et al., 2019
***O-GlcNAcylation***			
S366	MS/MS and mutagenesis	Promotes K63-linked ubiquitination of MAVS	Li et al., 2018
T321/S324/T328/S329/S300/S338/T342/S347	MS/MS and mutagenesis	Promotes K63-linked ubiquitination of MAVS	Song et al., 2019

*MAVS, mitochondrial antiviral signaling protein; MS, mass spectrometry; TRAF, tumor necrosis factor receptor-associated factor; PLK1, Polo-like kinase 1; TBK1, TANK binding kinase-1; IKK, IκB kinase; NLK, NEMO-like kinase.*

### RIG-I/MDA5 Ubiquitination

A series of E3 ubiquitin ligases catalyzing non-degradative lysine 63 (K63)-linked ubiquitination, including TRIM25, RIPLET, TRIM4, and MEX3C, were reported to induce MAVS activity by triggering ubiquitin-dependent RIG-I tetramerization through their CARDs (Okamoto et al., 2017). Interestingly, both covalently linked and unanchored ubiquitin chains were shown to promote RIG-I oligomerization. Unexpectedly, the same E3 ligase was reported to mediate either one or the other type of ubiquitination in independent studies (Oshiumi et al., 2009; Jiang et al., 2012). An explanation for these discrepant results was recently proposed by a co-immunoprecipitation (coIP)/MS-based proteomic study that revealed how the E3 ligase RIPLET can indeed mediate both types of ubiquitination depending on which type of E2 ubiquitin-conjugating enzyme has been bound (i.e. Ube2D3 for covalently linked ubiquitination, Ube2N for unanchored ubiquitination) (Shi et al., 2017). LC-MS/MS analyses have also contributed to identifying lysine residues 48, 96, and 172 in the CARDs of RIG-I that are ubiquitinated by RIPLET and required for MAVS activation (Shi et al., 2017).

K63-linked ubiquitination is also necessary for the oligomerization of MDA5. TRIM65 was identified by an LC-MS/MS-based interactome analysis as the E3 ligase responsible for this regulation by targeting the helicase domain of MDA5 at lysine 743 (Lang et al., 2017). Using a similar coIP/MS approach, TRIM65-mediated MDA5 ubiquitination was found to be stimulated by the interaction with the adaptor protein ARRDC4, whose expression is upregulated in infected cells (Meng et al., 2017).

Retinoic acid-inducible gene I/MDA5-mediated activation of MAVS needs to be tightly regulated in order to ensure the optimal activation and timely termination of innate antiviral response. To this aim, K63-linked ubiquitination of RIG-I and MDA5 is countered by a series of deubiquitinating enzymes (DUBs), such as USP3, USP21, and CYLD (Cui et al., 2014; Fan et al., 2014; Lin et al., 2016). Insights on how DUBs can be turned on during infection came from a study showing that CYLD activity is positively regulated by Syndecan-4 (SDC4), a proteoglycan whose expression is induced by type-I IFN. In a yeast two-hybrid screen, SDC4 was identified as a RIG-I interacting protein that promotes the binding of RIG-I with CYLD in order to decrease K63-linked ubiquitination (Lin et al., 2016).

Degradative ubiquitination also contributes to turning off the mitochondrial antiviral response by regulating the stability of RIG-I and MDA5. The E3 ligases STUB1, RNF122, RNF125, TRIM13, and TRIM40 (the first three identified by proteomic analyses) were shown to mediate lysine 27 (K27)- or lysine 48 (K48)-linked polyubiquitination of RIG-I/MDA5, which results in their proteasomal degradation (Arimoto et al., 2007; Narayan et al., 2014; Wang et al., 2016; Zhao et al., 2016, 2017; Zhou et al., 2018). How degradative ubiquitination is triggered upon RIG-I activation by non-degradative ubiquitination was investigated using an LC-MS/MS approach. This study showed that K63-linked polyubiquitination of RIG-I rapidly enhances its binding to a protein complex containing p97, UFD1, and NPL4, which in turn recruits the E3 ligase RNF125 to trigger K48-linked polyubiquitination of RIG-I and subsequent degradation (Hao et al., 2015).

Retinoic acid-inducible gene I stability is also controlled by autophagy, a catabolic process that plays an important role in the regulation of innate immune response by both degrading invading pathogens and contributing to the termination of the inflammatory response (Tal and Iwasaki, 2009). The autophagic degradation of RIG-I is regulated by leucine-rich repeat containing protein 25 (LRRC25), which binds to RIG-I when it is associated to the IFN-inducible ubiquitin-like protein 15 (ISG15). This protein complex promotes the interaction between RIG-I and the autophagic cargo receptor p62, which is responsible for the selective engulfment of RIG-I by autophagosomes (Du et al., 2018). Interestingly, during infection, this degradative mechanism is transiently counteracted by a different leucine-rich repeat containing protein, LRRC59, which binds to ISG15-associated RIG-I and inhibits the association between LRRC25 and RIG-I (Xian et al., 2019).

Conversely, degradative ubiquitination of MDA5 and RIG-I is negatively regulated by different mechanisms, such as deubiquitination, which is mediated by the DUB USP4 (Wang L. et al., 2013), and sumoylation, which prevents K48-linked ubiquitination of these proteins through the incorporation of the ubiquitin-like (UBL) modifier SUMO, as it was characterized by two-step immunoaffinity purification and LC-MS/MS analyses (Hu et al., 2017).

Recently, NLRP12, a protein of the NLR family, has been reported to negatively control the RLR signaling pathway by modulating both K63- and K48-linked ubiquitination of RIG-I, uncovering an important crosstalk between these innate immune receptor families. In detail, NLRP12 interacts with the ubiquitin ligase TRIM25, preventing RIG-I activation mediated by K63-linked ubiquitination, as well as with RNF125, enhancing K48-linked degradative ubiquitination of RIG-I (Chen et al., 2019). Of note, RIG-I degradation is also a mechanism by which the antiviral response is regulated by members of the lectin family, a different type of PRR that is activated by glycan PAMPs. In particular, the lectin Siglec-G (sialic-acid-binding immunoglobulin-like lectin G) promotes the interaction of the E3 ligase c-Cbl with RIG-I, which mediates its K48-linked ubiquitination in lysine 813 (Chen et al., 2013).

### MAVS Ubiquitination

Mitochondrial antiviral signaling is a direct target of both non-degradative and degradative ubiquitination in the course of antiviral response (Figure 2 and Table 1).

The E3 ligase TRIM31 has been identified as a regulator of MAVS aggregation via ubiquitination. During viral infection, TRIM31 is recruited to mitochondria, where it catalyzes K63-linked polyubiquitination of MAVS at lysine residues 10, 311, and 461 to facilitate the formation of prion-like aggregates (Liu et al., 2017). Interestingly, K63-linked ubiquitination of MAVS has been reported to be stimulated by O-GlcNAcylation, highlighting an unappreciated role of glucose metabolism in host innate immune (Li et al., 2018; Song et al., 2019). In detail, LC-MS/MS analysis identified serine 366 as the residue of MAVS where uridine diphosphate *N*-acetylglucosamine (UDP-GlcNAc) is linked by the O-GlcNAc transferase. Moreover, metabolomic analysis of viral infected macrophages revealed an increase of intermediate metabolites involved in hexosamine biosynthesis, the pathway responsible for the generation of UDP-GlcNAc. The functional relationship between RLRs and glucose metabolism was confirmed by the observation that lactate, the end product of glycolysis in anaerobic conditions, acts a negative regulator of MAVS signaling by preventing RIG-I–MAVS complex formation (Zhang et al., 2019). Interestingly, using a metabolomic approach, it was shown that RLR activation is accompanied by a decrease of glycolysis intermediates, including lactate, which facilitates MAVS-dependent production of type I IFN (Zhang et al., 2019). In line with these findings, inhibition of mitochondrial oxidative phosphorylation results in an impairment of MAVS-mediated induction of IFNs and inflammatory cytokines, further highlighting how antiviral response and mitochondria metabolism are strictly coordinated (Yoshizumi et al., 2017).

Non-degradative ubiquitination also favors the interaction of MAVS with downstream effectors. For example, viral infection induces the expression of the E3 ligase TRIM21, which binds MAVS to promote its K27-linked polyubiquitination at lysine 325 and its association with TBK1 (Xue et al., 2018). Moreover, TRIM proteins regulate the recruitment of IKKγ/NEMO to MAVS signalosome through their own ubiquitination. For example, upon viral infection, TRIM14 undergoes K63-linked polyubiquitination, which provides a platform for the binding of IKKγ/NEMO to MAVS on OMM (Zhou et al., 2014). The temporal kinetics of MAVS oligomerization is controlled by either DUBs or proteins containing UBL domains. YOD1, a deubiquitinase of the ovarian tumor family, is an MAVS interactor identified by coIP/MS that translocates to mitochondria upon viral infection to limit its activation through K63-linked deubiquitination (Liu et al., 2019). UBXN1 and FAF1, two proteins that contain ubiquitin-associated (UBA) or UBL domains, act as steric antagonists of MAVS. The UBA domain of UBXN1 competes with the TRAF3/6-binding sites of MAVS and interferes with its oligomerization (Wang P. et al., 2013). FAF1 forms aggregates through its UBL domain that negatively regulates MAVS by disrupting its association with TRIM31 (Dai et al., 2018).

K48-linked polyubiquitination represents a main mechanism to negatively regulate MAVS signaling by promoting its proteasomal degradation. Lysine 7 was identified as a K48-linked polyubiquitination site on MAVS in a high-resolution proteomic study aimed at investigating endogenous ubiquitination sites through the immuno-enrichment of the di-glycine-lysine remnants that are generated following tryptic digestion of ubiquitinated peptides (Wagner et al., 2012). More recently, it was shown that the mitochondrial E3 ligase MARCH5 binds to MAVS aggregates during viral infection and conjugates K48-linked polyubiquitin chains at lysine residues 7 and 500, leading to their proteasomal degradation (Yoo et al., 2015). Proteasomal degradation of MAVS is also triggered by: (i) the tumor suppressor E3 ligase VHL/Cullin 2 (Du et al., 2015), which promotes K48-linked polyubiquitination at lysine 420 residue, (ii) TRIM29, which mediates K11-linked polyubiquitination at lysine residues 371, 420, and 500 (Xing et al., 2018), and (iii) ITCH/AIP4, which targets lysine residues 371 and 420 with K48-linked polyubiquitination (You et al., 2009). In this regard, yeast two-hybrid screens identify poly rC binding protein 1 (PCBP1) and PCBP2 as positive regulators of the interaction between ITCH/AIP4 and MAVS, whose levels are increased upon viral infection (You et al., 2009). A similar role is played by the adaptor protein TAX1BP1, which is also required for promoting ITCH/AIP4-mediated ubiquitination of MAVS (Choi et al., 2017). Interestingly, TRIM25, an E3 ligase responsible for K63 ubiquitination of RIG-I, has also been identified as a MAVS binding partner using GeLC-MS/MS (1D SDS PAGE gel followed by LC-MS/MS). In particular, TRIM25 mediates K48-linked ubiquitination of MAVS at lysine residues 7 and 10, suggesting that the same E3 ligase can initially trigger the antiviral response and, later on, be responsible for its termination by inducing MAVS proteasomal degradation (Castanier et al., 2012). Other E3 ubiquitin ligases that catalyze K48 ubiquitination of MAVS are SMURF1, SMURF2, Ring Finger protein (RNF) 125, and GP78, but the ubiquitination sites have not been determined yet (Arimoto et al., 2007; Wang et al., 2012; Jacobs et al., 2014; Pan et al., 2014). Degradative ubiquitination is counteracted by DUBs, as shown for ovarian tumor family deubiquitinase 4 (OTUD4), whose expression is increased by IRF3/7 in a positive-feedback loop (Liuyu et al., 2019).

Ubiquitination can also trigger the autophagic degradation of MAVS. The E3 ubiquitin RNF34 promotes K27/K29-linked polyubiquitination on MAVS at lysine residues 297, 311, 348, and 362. Ubiquitinated MAVS-enriched mitochondria are then recognized by the autophagy receptor NDP52 and delivered to autophagosomes for degradation (He et al., 2019). Similarly, the IFN-induced Tetherin (also known as BST2) recruits the E3 ligase MARCH8 to mediate K27-linked ubiquitination of MAVS at lysine 7 and subsequent NDP52-dependent autophagic degradation, which is important to prevent excessive activation of RLR signaling (Jin et al., 2017).

### RIG-I/MDA5 Phosphorylation

Protein phosphorylation is a second main type of PTM that controls MAVS signaling during viral infection (Figure 2). This regulation is important to modulate both the binding of RIG-I/MDA5 to MAVS and the recruitment of downstream effectors to assemble the MAVS signalosome (Oshiumi et al., 2016).

On one hand, tyrosine kinases of the SRC family have been shown to stimulate RIG-I activity. CoIP/MS analyses identified LYN as a binding partner of both RIG-I and MAVS in macrophages upon treatment with synthetic analogs of double-stranded RNA (Lim et al., 2015). In this condition, LYN phosphorylates RIG-I at tyrosine 396 and promotes its oligomerization. c-SRC was also observed to stimulate RIG-I, but it acts in an indirect manner (Lee et al., 2018). In fact, c-SRC phosphorylates TRIM25 at tyrosine 278, thus stimulating K63-linked ubiquitination and oligomerization of RIG-I.

On the other hand, RIG-I/MDA5 phosphorylation in serine and threonine is mainly associated to the inhibition of their activity in resting conditions. The negative roles of this type of phosphorylation were highlighted by an LC-MS/MS analysis of phosphorylated peptides differentially present in ubiquitinated and non-ubiquitinated RIG-I. This study identified, among others, serine 8 and threonine 170 as phosphorylated sites that negatively regulate the ubiquitination on lysine 172 by preventing the binding of TRIM25 (Gack et al., 2010; Nistal-Villán et al., 2010). These amino acids are phosphorylated by protein kinase C-α (PKC-α) and PKC-β (Maharaj et al., 2012). Inhibitory phosphorylations were also identified at threonine 770, serine 854, and serine 855 at the C-term domain RIG-I, residues that are phosphorylated by casein kinase II (CK2), and at serine 828 of MDA5, which is carried out by RIO kinase 3 (RIOK3) (Sun et al., 2011; Takashima et al., 2015). In both cases, phosphorylation prevents the oligomerization of these sensor proteins.

Early during viral infection, serine/threonine phosphorylation levels of RIG-I/MDA5 are decreased by PP1α and PP1γ, two protein phosphatases that have been identified through a phosphatome RNAi screen (Wies et al., 2013). A combination of RNA interference, yeast two-hybrid, and APEX2 proximity labeling-based MS approaches also identified PPP6C as a phosphatase that controls RIG-I activity. In particular, PPP6C forms a complex with WHIP and TRIM14, which mediate the binding of PPP6C to ubiquitinated RIG-I and MAVS, respectively (Tan et al., 2017). At later stages of infection, the inhibitory phosphorylations are re-established and contribute to turning off MAVS signaling. This event is regulated by death associated protein kinase 1 (DAPK1), whose activation is triggered by RIG-I signaling as a negative-feedback mechanism (Willemsen et al., 2017).

### MAVS Phosphorylation

Various phosphorylations of MAVS have also been characterized with either positive or negative effects on its function (Figure 2 and Table 1).

Stimulatory phosphorylations were identified by LC-MS/MS analyses at serine residues 442, 444, and 44 in the C terminus of MAVS, which are phosphorylated by TBK1 and IKKβ upon viral infection. This modification is needed to recruit IRF3 on MAVS signalosome and facilitate its phosphorylation by TBK1 (Liu et al., 2015). Phosphorylation-driven formation of MAVS signaling complex is countered by PPM1A (protein phosphatase magnesium-dependent 1A, also known as PP2Cα), which targets both MAVS and TBK1/IKKε for dephosphorylation (Xiang et al., 2016).

c-ABL has been reported to phosphorylate MAVS at tyrosine 9, promoting the formation of MAVS/TRAF3/TRAF6 complex and the activation of the antiviral response (Song et al., 2010). Recently, the role of c-ABL-mediated phosphorylation of MAVS has been further investigated in the context of microglia inflammation, where this kinase promotes MAVS activity by disrupting its interaction with the autophagosome protein LC3 and preventing autophagy-mediated MAVS degradation (Cheng et al., 2017). Additional activating phosphorylations of MAVS have been identified by proteomic approaches, but the molecular mechanism underlying their effect remains to be elucidated. For example, a quantitative analysis of phospho-peptides using iTRAQ labeling identified serine residues 328 and 330 in mouse MAVS sequence as required for the stimulation of IRF3 and NF-κB activity (Kandasamy et al., 2016).

Conversely, phosphorylation of threonine 234 and serine 233 of MAVS was shown to inhibit the antiviral response by serving as a docking site for the recruitment of mitotic kinase Polo-like kinase 1 (PLK1). The PLK1–MAVS interaction, identified through a yeast two-hybrid screen, interferes with the binding of MAVS with TRAF3 and attenuates IFN signaling during viral infection as well as during the G2/M phase of the cell cycle (Vitour et al., 2009). Recently, an LC-MS/MS study showed that MAVS phosphorylation could also prime MAVS for proteasomal degradation. This is mediated by the NEMO-like kinase (NLK), which targets MAVS at serine residues 121, 212, 258, and 329 (Li et al., 2019).

### RIG-I Acetylation

A third type of PTM that has been shown to regulate MAVS signaling is protein acetylation. LC-MS/MS analysis identified lysine residues 858 and 909 as acetylated residues of RIG-I that contribute to preventing its oligomerization in resting conditions. The acetyltransferases responsible for this PTM remain to be characterized, while HDAC6 was identified as the deacetylase required for removal of these acetyl groups during viral infection to allow RIG-I activation (Choi et al., 2016; Liu et al., 2016).

## Regulation of MAVS Signaling by Protein–Protein Interaction

### Cellular Protein Interactions

**FIGURE 3:**
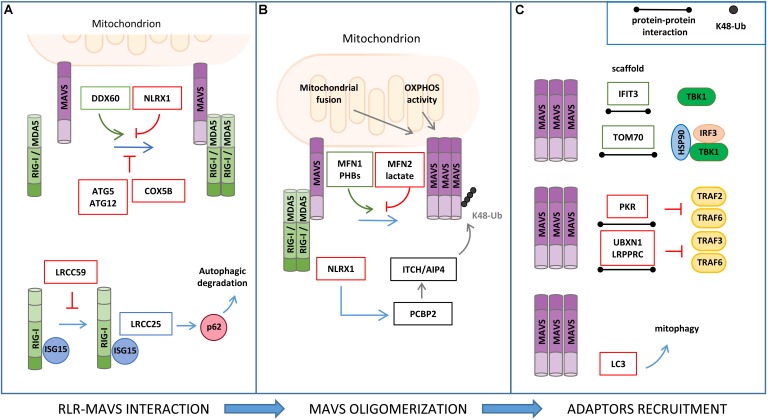
Regulation of RLR–MAVS signaling by protein–protein interaction. **(A)** RIG-I/MDA5 interaction with MAVS is facilitated by DDX60 and inhibited by NLRX1, ATG5/ATG12 complex, and COX5B (upper panel). ISG15 association to RIG-I promotes leucine-rich repeat containing protein 25 (LRRC25) interaction and p62-mediated autophagic degradation of RIG-I, which is inhibited by LRCC59 (lower panel). **(B)** Mitochondrial fusion and oxidative phosphorylation (OXPHOS) are required for efficient MAVS signaling. In particular, MFN1 and PHB1 and PHB2 act as positive regulators of MAVS signaling, while MFN2, lactate, and NLRX1 negatively regulate MAVS activity. **(C)** Interferon Induced Protein With Tetratricopeptide Repeats 3 (IFIT3) and TOM70 interact with MAVS and function as a scaffold for the recruitment of downstream effectors; protein kinase R (PKR), UBXN1, and LRPPRC interact with MAVS, inhibiting TRAF association (upper and middle panels); LC3–MAVS interaction promotes mitochondrial degradation by mitophagy (lower panels).

In addition to PTMs, proteomic analyses have provided a large set of information on how MAVS signaling is regulated by direct protein–protein interactions with cellular and viral factors (Figure 3). Interactomics studies were carried out using either antibody-based affinity pull-down combined with MS analyses, which allows the characterization of protein complexes in a physiological context but favoring stable associations, or yeast two-hybrid assays, which also detect transient or labile interactions but assessed in non-physiological cell systems (Mehta and Trinkle-Mulcahy, 2016).

Various studies have reported that ISGs that are induced upon MAVS activation may establish a positive-feedback loop to potentiate the antiviral signaling at multiple steps (Crosse et al., 2018). For example, the ISG protein DDX60 is an RNA helicase that, upon binding with double-strand RNA, associates with RIG-I, MDA5, and LGP2, and increases MAVS activation (Miyashita et al., 2011). The shorter isoform of zinc-finger antiviral protein (S-ZAP) is an ISG that associates with RIG-I to promote its oligomerization and activity (Hayakawa et al., 2011). Interferon Induced Protein With Tetratricopeptide Repeats 3 (IFIT3) localizes to mitochondria, where it functions as a scaffold to facilitate the interaction of MAVS with TBK1 (Liu et al., 2011). The dsRNA-dependent protein kinase R (PKR) is upregulated by type I and type III IFNs and induces MAVS signaling by interacting with TRAF2 and TRAF6 to promote NF-κB activation (Gil et al., 2004).

The cytoskeletal network is another source of proteins that were found to stimulate the antiviral response by interacting with components of MAVS signaling. Focal adhesion kinase (FAK) is an actin-associated tyrosine kinase that binds to MAVS in a viral infection-dependent manner and potentiates its signaling independently of its kinase activity (Bozym et al., 2012). Two microtubule proteins, Rho guanine nucleotide exchange factor 2 (GEF-H1) and HAUS augmin-like complex subunit 8 (HAUS8), also act as positive regulators of the RLR pathway by stimulating TBK1 activity and MAVS ubiquitination, respectively (Chiang et al., 2014; He et al., 2018).

A recent study in which global RNA interference analyses were combined to experimentally and computationally derive interactome data has provided an in-depth view of the RIG-I protein interaction network and identified new processes that influence the host response to viral RNA, including the unfolded protein response, WNT/β-catenin signaling, and RNA metabolism. The role of K-Homology Splicing Regulatory Protein (KHSRP), one of the best hits obtained from this screening, was further characterized, showing that its interaction with RIG-I is required to maintain the receptor in an inactive state in resting conditions (Soonthornvacharin et al., 2017).

The mitochondrial resident factors NLRX1, LRPPRC, MFN1, MFN2, TOM70, PHB1/2, and LONP1 have been described to interact with MAVS and regulate the antiviral signaling.

NLRX1 (nucleotide-binding domain and leucine-rich repeat containing family member) was the first mitochondrial protein identified as a negative regulator of MAVS (Allen et al., 2011). NLRX1 interacts with MAVS via its CARD and interferes with the binding of MAVS with the upstream partners RIG-I and MDA5. However, the role of NLRX1 in MAVS signaling has long been debated. NLRX1-deficient mice exhibit unaltered antiviral and inflammatory responses to Sendai virus, influenza A virus, or synthetic analogs of double-stranded RNA injection when compared to wild-type mice (Rebsamen et al., 2011; Soares et al., 2013). In addition, NLRX1 may also play a positive role in innate immune signaling mediated by NF-κB through the generation of ROS, which is supported by the observations that NLRX1 is mainly localized to the mitochondrial matrix and interacts with UQCRC2, a protein of the respiratory chain complex III (Arnoult et al., 2009). More recently, the mechanism by which NLRX1 negatively regulates the antiviral response has been refined by studying hepatitis C virus (HCV) infection. In this context, NLRX1 functions as a bridging protein between MAVS and PCBP2, thus promoting the ITCH/AIP4-mediated K48-linked polyubiquitination and degradation of MAVS (Qin et al., 2017).

LRPPRC, a protein known to regulate mitochondrial RNA stability, has been characterized as a negative regulator of the mitochondrial-mediated antiviral immunity during HCV infection. In particular, LRPPRC interacts with MAVS and inhibits its signaling by preventing the association with TRAF3 and TRAF6 (Refolo et al., 2019).

The mitochondrial fusion protein MFN2 has been identified as a negative regulator of MAVS-mediated antiviral signaling through an LC-MS/MS-based interactome analysis. MFN2 binds to MAVS, preventing its oligomerization and the propagation of the downstream antiviral response (Yasukawa et al., 2009). This MFN2 function is distinct from its role in mitochondrial dynamics. In fact, the inhibition of mitochondrial fusion by the concomitant downregulation of MFN1 and MFN2 expression (Koshiba et al., 2011), as well as that of OPA1 (Yoshizumi et al., 2017), leads to the repression of MAVS activity, which is associated to a dissipation of mitochondrial membrane potential. Moreover, MFN1 was shown to physically interact with MAVS and promote RIG-I-mediated MAVS oligomerization, suggesting also a direct involvement of MFN1 in the induction of the mitochondrial antiviral response (Onoguchi et al., 2010).

Analysis of MAVS interacting proteins upon mitochondrial enrichment and GeLC-MS/MS identified the mitochondrial protein TOM70 (translocase of outer membrane 70) as a novel component of the MAVS signalosome. In detail, Tom70 acts as a promoting factor of the antiviral response by functioning as a scaffold for the recruitment of both IRF3 and TBK1, in association with HSP90, to MAVS (Liu et al., 2010).

A positive role of the inner mitochondrial membrane (IMM) proteins PROHIBITIN 1 (PHB1) and PHB2 in MAVS signaling has recently emerged in a proteomic study aimed at characterizing the function of the coiled coil domains of these mitochondrial scaffold proteins. Upon viral infection, PHBs were shown to form a complex with MAVS oligomers, which also includes the AAA(+) ATPase proteins Caseinolytic Peptidase B (CLPB) Homolog and ATPase Family AAA Domain Containing 3A (ATAD3A), thus establishing a bridge between the OMM and IMM that is required for the efficient activation of the RIG-I signaling pathway (Yoshinaka et al., 2019).

The mitochondrial protease LONP1 has been identified as a positive regulator of MAVS in a coIP/MS-based analysis from purified MAM, a study that was performed using surfactants hydrolyzable at low pH to reduce detergent interference in MS (Horner et al., 2015). However, the molecular mechanism underlying this regulation remains to be characterized.

Mitochondrial antiviral signaling activity is also regulated by autophagy through protein–protein interaction. MAVS directly binds to the autophagosome protein LC3 through an LC3-interaction region (LIR) at the MAVS N-terminal region. Through this interaction, MAVS was reported to function as a mitophagy receptor for the engulfment of mitochondria that are damaged upon excessive activation of antiviral signaling (Sun et al., 2016). Moreover, the autophagy proteins ATG5–ATG12 were found to downregulate RIG-I signaling by interacting with the CARDs of both RIG-I and MAVS (Jounai et al., 2007). Interestingly, the mitochondrial protein COX5B has been shown to act in concert with ATG5 in the suppression of MAVS activity both through direct protein interaction and, indirectly, by decreasing ROS levels (Zhao et al., 2012).

The identification of gC1qR (receptor for globular head domain of complement component C1q) and Sorting nexin 8 (SNX8) as MAVS interacting proteins that regulate the antiviral response suggests a role of other signaling pathways to MAVS activity that remain to be contextualized (Xu et al., 2009; Guo et al., 2019).

### Viral Protein Interactions

Viruses have evolved different mechanisms to inhibit MAVS activity as part of their immune evasion strategies. Viral proteins are able to interfere with the binding of MAVS to the upstream RLR sensors. The non-structural protein NS4A of Dengue and Zika viruses binds to the CARD of MAVS, preventing RIG-I/MDA5 accessibility and hampering RLR signal transduction (He et al., 2016; Ma et al., 2018). Zika NS3 also contributes to the inhibition of MAVS activation, as demonstrated in an interactome study where NS3 was found to bind and inhibit the function of 14-3-3ε, a docking protein required for the translocation of RIG-I/MDA5 to mitochondria (Riedl et al., 2019).

We have recently characterized the interactome of HCV NS5A using an HCV replicon cell system in which a double tag was inserted in the viral genome in a region of NS5A that did not alter its ability to support viral replication. NS5A coIP was carried out using *n*-dodecyl β-D-maltoside as a detergent to preserve the integrity of mitochondrial membrane complexes. We found that HCV NS5A interacts with the mitochondrial protein LRPPRC and represses the antiviral response by promoting LRPPRC association with MAVS, which results in a reduced association of MAVS with TRAF proteins (Refolo et al., 2019).

A peculiar mechanism to elude MAVS activity has been described for human immunodeficiency virus 1 (HIV-1) in dendritic cells. In these cells, the RNA helicase DDX3 acts as an intracellular sensor of abortive HIV-1 RNAs that stimulates MAVS activity by promoting its interaction with TRAF3. HIV-1 is able to block this pathway through the HIV-1 envelope protein GP120, which, by binding to the C-type lectin receptor DC-SIGN, activates PLK1 to interfere with the formation of MAVS–TRAF3 complex (Gringhuis et al., 2017).

Finally, a large series of viral proteases induces the proteolytic cleavage of MAVS to release it from the outer membrane of mitochondria. These include: HCV NS3/4A viral protease, which cleaves MAVS at cysteine 508 (Li et al., 2005); hepatitis A virus (HAV) 3ABC precursor of 3Cpro cysteine protease (Yang et al., 2007); 3Cpro cysteine protease of coxsackievirus B3 (CVB3), which cleaves MAVS at Q148 within the proline-rich region (Mukherjee et al., 2011); and enterovirus 71 (EV71) 2A protease (2Apro), which cleaves MAVS on multiple residues, at Gly209, Gly251, and Gly265 (Wang B. et al., 2013). A different inhibitory mechanism has been described for the influenza A virus PB1-F2 protein and the human cytomegalovirus (HCMV) glycoprotein US9, which interact with MAVS and inhibit its activity by dissipating the mitochondrial membrane potential (Varga et al., 2012; Choi et al., 2018).

## Concluding Remarks

The mitochondrial proteome is a highly dynamic entity that undergoes profound changes in response to a variety of stress conditions in the attempt to mount an adaptive response or, when not feasible, to trigger programmed cell death. In this review, we discussed recent findings describing how the host cell response to RNA virus infection is centered on the mitochondrial outer membrane. In this subcellular compartment, MAVS signalosome is rapidly assembled to recruit key components of IRFs and NF-κB signaling pathways and induces the expression of hundreds of genes with antiviral properties. In particular, we highlighted how proteomic approaches have contributed to the characterization of protein networks and their PTMs that regulate MAVS signaling. In addition, for its unbiased characteristics, proteomics has provided several new insights on the crosstalk of the mitochondrial antiviral response with other PPRs, such as NLR and lectins, or other cellular processes, such as glucose metabolism. The key contribution of proteomic studies to unraveling the complexity of the antiviral response is expected to have an important impact on the development of novel therapeutic strategies aimed at suppressing viral infection and enhancing immune responses.

## Author Contributions

GR, GF, and FC wrote the text and prepared the figures. TV and MP contributed to literature search and revised the manuscript and discussion.

## Conflict of Interest

The authors declare that the research was conducted in the absence of any commercial or financial relationships that could be construed as a potential conflict of interest.

## References

[B1] Allen I. C. Moore C. B. Schneider M. Lei Y. Davis B. K. Scull M. A. (2011). NLRX1 protein attenuates inflammatory responses to infection by interfering with the RIG-I-MAVS and TRAF6-NF-κB signaling pathways. *Immunity* 34 854–865. 10.1016/j.immuni.2011.03.026 21703540 PMC3166771

[B2] Arimoto K. Takahashi H. Hishiki T. Konishi H. Fujita T. Shimotohno K. (2007). Negative regulation of the RIG-I signaling by the ubiquitin ligase RNF125. *Proc. Natl. Acad. Sci. U.S.A.* 104 7500–7505. 10.1073/pnas.0611551104 17460044 PMC1863485

[B3] Arnoult D. Soares F. Tattoli I. Castanier C. Philpott D. J. Girardin S. E. (2009). An N-terminal addressing sequence targets NLRX1 to the mitochondrial matrix. *J. Cell Sci.* 122 3161–3168. 10.1242/jcs.051193 19692591 PMC2871076

[B4] Bozym R. A. Delorme-Axford E. Harris K. Morosky S. Ikizler M. Dermody T. S. (2012). Focal adhesion kinase is a component of antiviral RIG-I-like receptor signaling. *Cell Host Microbe* 11 153–166. 10.1016/j.chom.2012.01.008 22341464 PMC3995454

[B5] Brisse M. Ly H. (2019). Comparative structure and function analysis of the RIG-I-Like receptors: RIG-I and MDA5. *Front. Immunol.* 10:01586. 10.3389/fimmu.2019.01586 31379819 PMC6652118

[B6] Brubaker S. W. Bonham K. S. Zanoni I. Kagan J. C. (2015). Innate immune pattern recognition: a cell biological perspective. *Annu. Rev. Immunol.* 33 257–290. 10.1146/annurev-immunol-032414-112240 25581309 PMC5146691

[B7] Brubaker S. W. Gauthier A. E. Mills E. W. Ingolia N. T. Kagan J. C. (2014). A bicistronic MAVS transcript highlights a class of truncated variants in antiviral immunity. *Cell* 156 800–811. 10.1016/j.cell.2014.01.021 24529381 PMC3959641

[B8] Bruns A. M. Horvath C. M. (2015). LGP2 synergy with MDA5 in RLR-mediated RNA recognition and antiviral signaling. *Cytokine* 74 198–206. 10.1016/j.cyto.2015.02.010 25794939 PMC4475439

[B9] Castanier C. Zemirli N. Portier A. Garcin D. Bidère N. Vazquez A. (2012). MAVS ubiquitination by the E3 ligase TRIM25 and degradation by the proteasome is involved in type I interferon production after activation of the antiviral RIG-I-like receptors. *BMC Biol.* 10:44. 10.1186/1741-7007-10-44 22626058 PMC3372421

[B10] Chen W. Han C. Xie B. Hu X. Yu Q. Shi L. (2013). Induction of Siglec-G by RNA viruses inhibits the innate immune response by promoting RIG-I degradation. *Cell* 152 467–478. 10.1016/j.cell.2013.01.011 23374343

[B11] Chen S.-T. Chen L. Lin D. S.-C. Chen S.-Y. Tsao Y.-P. Guo H. (2019). NLRP12 regulates anti-viral RIG-I activation via interaction with TRIM25. *Cell Host Microbe* 25 602.e7–616.e7. 10.1016/j.chom.2019.02.013 30902577 PMC6459718

[B12] Cheng J. Liao Y. Xiao L. Wu R. Zhao S. Chen H. (2017). Autophagy regulates MAVS signaling activation in a phosphorylation-dependent manner in microglia. *Cell Death Differ.* 24 276–287. 10.1038/cdd.2016.121 28141795 PMC5299710

[B13] Chiang H.-S. Zhao Y. Song J.-H. Liu S. Wang N. Terhorst C. (2014). GEF-H1 controls microtubule-dependent sensing of nucleic acids for antiviral host defenses. *Nat. Immunol.* 15 63–71. 10.1038/ni.2766 24270516 PMC4066330

[B14] Choi H. J. Park A. Kang S. Lee E. Lee T. A. Ra E. A. (2018). Human cytomegalovirus-encoded US9 targets MAVS and STING signaling to evade type I interferon immune responses. *Nat. Commun.* 9:125. 10.1038/s41467-017-02624-8 29317664 PMC5760629

[B15] Choi S. J. Lee H.-C. Kim J.-H. Park S. Y. Kim T.-H. Lee W.-K. (2016). HDAC6 regulates cellular viral RNA sensing by deacetylation of RIG-I. *EMBO J.* 35 429–442. 10.15252/embj.201592586 26746851 PMC4755110

[B16] Choi Y. B. Shembade N. Parvatiyar K. Balachandran S. Harhaj E. W. (2017). TAX1BP1 restrains virus-induced apoptosis by facilitating itch-mediated degradation of the mitochondrial adaptor MAVS. *Mol. Cell. Biol.* 37:e00422-16. 10.1128/mcb.00422-16 27736772 PMC5192085

[B17] Crosse K. M. Monson E. A. Beard M. R. Helbig K. J. (2018). Interferon-stimulated genes as enhancers of antiviral innate immune signaling. *J. Innate Immun.* 10 85–93. 10.1159/000484258 29186718 PMC5969054

[B18] Cui J. Song Y. Li Y. Zhu Q. Tan P. Qin Y. (2014). USP3 inhibits type I interferon signaling by deubiquitinating RIG-I-like receptors. *Cell Res.* 24 400–416. 10.1038/cr.2013.170 24366338 PMC3975496

[B19] Dai T. Wu L. Wang S. Wang J. Xie F. Zhang Z. (2018). FAF1 regulates antiviral immunity by inhibiting MAVS but is antagonized by phosphorylation upon viral infection. *Cell Host Microbe* 24 776.e5–790.e5. 10.1016/j.chom.2018.10.006 30472208

[B20] Dixit E. Boulant S. Zhang Y. Lee A. S. Y. Odendall C. Shum B. (2010). Peroxisomes are signaling platforms for antiviral innate immunity. *Cell* 141 668–681. 10.1016/j.cell.2010.04.018 20451243 PMC3670185

[B21] Du J. Zhang D. Zhang W. Ouyang G. Wang J. Liu X. (2015). pVHL negatively regulates antiviral signaling by targeting MAVS for proteasomal degradation. *J. Immunol.* 195 1782–1790. 10.4049/jimmunol.1500588 26179906

[B22] Du Y. Duan T. Feng Y. Liu Q. Lin M. Cui J. (2018). LRRC25 inhibits type I IFN signaling by targeting ISG15-associated RIG-I for autophagic degradation. *EMBO J.* 37 351–366. 10.15252/embj.201796781 29288164 PMC5793803

[B23] Fan Y. Mao R. Yu Y. Liu S. Shi Z. Cheng J. (2014). USP21 negatively regulates antiviral response by acting as a RIG-I deubiquitinase. *J. Exp. Med.* 211 313–328. 10.1084/jem.20122844 24493797 PMC3920558

[B24] Franchi L. Muñoz-Planillo R. Núñez G. (2012). Sensing and reacting to microbes through the inflammasomes. *Nat. Immunol.* 13 325–332. 10.1038/ni.2231 22430785 PMC3449002

[B25] Gack M. U. Nistal-Villan E. Inn K.-S. Garcia-Sastre A. Jung J. U. (2010). Phosphorylation-mediated negative regulation of RIG-I antiviral activity. *J. Virol.* 84 3220–3229. 10.1128/jvi.02241-09 20071582 PMC2838087

[B26] Gil J. Garcia M. A. Gomez-Puertas P. Guerra S. Rullas J. Nakano H. (2004). TRAF family proteins link PKR with NF- B activation. *Mol. Cell. Biol.* 24 4502–4512. 10.1128/mcb.24.10.4502-4512.2004 15121867 PMC400457

[B27] Gringhuis S. I. Hertoghs N. Kaptein T. M. Zijlstra-Willems E. M. Sarrami-Fooroshani R. Sprokholt J. K. (2017). HIV-1 blocks the signaling adaptor MAVS to evade antiviral host defense after sensing of abortive HIV-1 RNA by the host helicase DDX3. *Nat. Immunol.* 18 225–235. 10.1038/ni.3647 28024153

[B28] Guo W. Wei J. Zhong X. Zang R. Lian H. Hu M.-M. (2019). SNX8 modulates the innate immune response to RNA viruses by regulating the aggregation of VISA. *Cell. Mol. Immunol.* [Epub ahead of print]. 31511639 10.1038/s41423-019-0285-2 PMC7784681

[B29] Hao Q. Jiao S. Shi Z. Li C. Meng X. Zhang Z. (2015). A non-canonical role of the p97 complex in RIG-I antiviral signaling. *EMBO J.* 34 2903–2920. 10.15252/embj.201591888 26471729 PMC4687688

[B30] Hayakawa S. Shiratori S. Yamato H. Kameyama T. Kitatsuji C. Kashigi F. (2011). ZAPS is a potent stimulator of signaling mediated by the RNA helicase RIG-I during antiviral responses. *Nat. Immunol.* 12 37–44. 10.1038/ni.1963 21102435

[B31] Hayashi T. Rizzuto R. Hajnoczky G. Su T.-P. (2009). MAM: more than just a housekeeper. *Trends Cell Biol.* 19 81–88. 10.1016/j.tcb.2008.12.002 19144519 PMC2750097

[B32] He T.-S. Chen T. Wang D.-D. Xu L.-G. (2018). HAUS8 regulates RLR-VISA antiviral signaling positively by targeting VISA. *Mol. Med. Rep.* 18 2458–2466. 10.3892/mmr.2018.9171 29916539

[B33] He X. Zhu Y. Zhang Y. Geng Y. Gong J. Geng J. (2019). RNF34 functions in immunity and selective mitophagy by targeting MAVS for autophagic degradation. *EMBO J.* 38 e100978. 10.15252/embj.2018100978 31304625 PMC6627233

[B34] He Z. Zhu X. Wen W. Yuan J. Hu Y. Chen J. (2016). Dengue virus subverts host innate immunity by targeting adaptor protein MAVS. *J. Virol.* 90 7219–7230. 10.1128/jvi.0022116 27252539 PMC4984625

[B35] Horner S. M. Liu H. M. Park H. S. Briley J. Gale M. (2011). Mitochondrial-associated endoplasmic reticulum membranes (MAM) form innate immune synapses and are targeted by hepatitis C virus. *Proc. Natl. Acad. Sci. U.S.A.* 108 14590–14595. 10.1073/pnas.1110133108 21844353 PMC3167523

[B36] Horner S. M. Wilkins C. Badil S. Iskarpatyoti J. Gale M. (2015). Proteomic analysis of mitochondrial-associated ER membranes (MAM) during RNA virus infection reveals dynamic changes in protein and organelle trafficking. *PLoS One* 10:e0117963. 10.1371/journal.pone.0117963 25734423 PMC4348417

[B37] Hou F. Sun L. Zheng H. Skaug B. Jiang Q.-X. Chen Z. J. (2011). MAVS forms functional prion-like aggregates to activate and propagate antiviral innate immune response. *Cell* 146 448–461. 10.1016/j.cell.2011.06.041 21782231 PMC3179916

[B38] Hu M.-M. Liao C.-Y. Yang Q. Xie X.-Q. Shu H.-B. (2017). Innate immunity to RNA virus is regulated by temporal and reversible sumoylation of RIG-I and MDA5. *J. Exp. Med.* 214 973–989. 10.1084/jem.20161015 28250012 PMC5379974

[B39] Jacobs J. L. Zhu J. Sarkar S. N. Coyne C. B. (2014). Regulation of mitochondrial antiviral signaling (MAVS) expression and signaling by the mitochondria-associated endoplasmic reticulum membrane (MAM) protein Gp78. *J. Biol. Chem.* 289 1604–1616. 10.1074/jbc.M113.520254 24285545 PMC3894340

[B40] Jiang X. Kinch L. N. Brautigam C. A. Chen X. Du F. Grishin N. V. (2012). Ubiquitin-induced oligomerization of the RNA sensors RIG-I and MDA5 activates antiviral innate immune response. *Immunity* 36 959–973. 10.1016/j.immuni.2012.03.022 22705106 PMC3412146

[B41] Jin S. Tian S. Luo M. Xie W. Liu T. Duan T. (2017). Tetherin suppresses type I interferon signaling by Targeting MAVS for NDP52-mediated selective autophagic degradation in human cells. *Mol. Cell* 68 308.e4–322.e4. 10.1016/j.molcel.2017.09.005 28965816

[B42] Jounai N. Takeshita F. Kobiyama K. Sawano A. Miyawaki A. Xin K.-Q. (2007). The Atg5 Atg12 conjugate associates with innate antiviral immune responses. *Proc. Natl. Acad. Sci. U.S.A.* 104 14050–14055. 10.1073/pnas.0704014104 17709747 PMC1955809

[B43] Kandasamy R. K. Vladimer G. I. Snijder B. Müller A. C. Rebsamen M. Bigenzahn J. W. (2016). A time-resolved molecular map of the macrophage response to VSV infection. *NPJ Syst. Biol. Appl.* 2 16027. 10.1038/npjsba.2016.27 28725479 PMC5516859

[B44] Kato H. Takeuchi O. Sato S. Yoneyama M. Yamamoto M. Matsui K. (2006). Differential roles of MDA5 and RIG-I helicases in the recognition of RNA viruses. *Nature* 441 101–105. 10.1038/nature04734 16625202

[B45] Kawai T. Akira S. (2011). Toll-like Receptors and Their Crosstalk with Other Innate Receptors in Infection and Immunity. *Immunity* 34 637–650. 10.1016/j.immuni.2011.05.006 21616434

[B46] Komuro A. Horvath C. M. (2006). RNA- and Virus-Independent Inhibition of Antiviral Signaling by RNA Helicase LGP2. *J. Virol.* 80 12332–12342. 10.1128/jvi.01325-06 17020950 PMC1676302

[B47] Koshiba T. Yasukawa K. Yanagi Y. Kawabata S. I. (2011). Mitochondrial membrane potential is required for MAVS-mediated antiviral signaling. *Sci. Signal* 4 ra7. 10.1126/scisignal.2001147 21285412

[B48] Kowalinski E. Lunardi T. McCarthy A. A. Louber J. Brunel J. Grigorov B. (2011). Structural basis for the activation of innate immune pattern-recognition receptor RIG-I by viral RNA. *Cell* 147 423–435. 10.1016/j.cell.2011.09.039 22000019

[B49] Lang X. Tang T. Jin T. Ding C. Zhou R. Jiang W. (2017). TRIM65-catalized ubiquitination is essential for MDA5-mediated antiviral innate immunity. *J. Exp. Med.* 214 459–473. 10.1084/jem.20160592 28031478 PMC5294850

[B50] Lazear H. M. Schoggins J. W. Diamond M. S. (2019). Shared and distinct functions of type I and type III interferons. *Immunity* 50 907–923. 10.1016/j.immuni.2019.03.025 30995506 PMC6839410

[B51] Lee N.-R. Choi J.-Y. Yoon I.-H. Lee J. K. Inn K.-S. (2018). Positive regulatory role of c-Src-mediated TRIM25 tyrosine phosphorylation on RIG-I ubiquitination and RIG-I-mediated antiviral signaling pathway. *Cell. Immunol.* 332 94–100. 10.1016/j.cellimm.2018.08.004 30100205

[B52] Li S.-Z. Shu Q.-P. Song Y. Zhang H.-H. Liu Y. Jin B.-X. (2019). Phosphorylation of MAVS/VISA by Nemo-like kinase (NLK) for degradation regulates the antiviral innate immune response. *Nat. Commun.* 10:3233. 10.1038/s41467-019-11258-x 31324787 PMC6642205

[B53] Li X.-D. Sun L. Seth R. B. Pineda G. Chen Z. J. (2005). Hepatitis C virus protease NS3/4A cleaves mitochondrial antiviral signaling protein off the mitochondria to evade innate immunity. *Proc. Natl. Acad. Sci. U.S.A.* 102 17717–17722. 10.1073/pnas.0508531102 16301520 PMC1308909

[B54] Li T. Li X. Attri K. S. Liu C. Li L. Herring L. E. (2018). O-GlcNAc transferase links glucose metabolism to MAVS-mediated antiviral innate immunity. *Cell Host Microbe* 24 791.e6–803.e6. 10.1016/j.chom.2018.11.001 30543776 PMC6296827

[B55] Lim Y. J. Koo J. E. Hong E.-H. Park Z.-Y. Lim K.-M. Bae O.-N. (2015). A Src-family-tyrosine kinase, Lyn, is required for efficient IFN-β expression in pattern recognition receptor, RIG-I, signal pathway by interacting with IPS-1. *Cytokine* 72 63–70. 10.1016/j.cyto.2014.12.008 25585356

[B56] Lin W. Zhang J. Lin H. Li Z. Sun X. Xin D. (2016). Syndecan-4 negatively regulates antiviral signalling by mediating RIG-I deubiquitination via CYLD. *Nat. Commun.* 7:11848. 10.1038/ncomms11848 27279133 PMC4906230

[B57] Liu B. Zhang M. Chu H. Zhang H. Wu H. Song G. (2017). The ubiquitin E3 ligase TRIM31 promotes aggregation and activation of the signaling adaptor MAVS through Lys63-linked polyubiquitination. *Nat. Immunol.* 18 214–224. 10.1038/ni.3641 27992402

[B58] Liu C. Huang S. Wang X. Wen M. Zheng J. Wang W. (2019). The otubain YOD1 suppresses aggregation and activation of the signaling adaptor MAVS through Lys63-linked deubiquitination. *J. Immunol.* 202 2957–2970. 10.4049/jimmunol.1800656 30952814

[B59] Liu H. M. Jiang F. Loo Y. M. Hsu S. Hsiang T.-Y. Marcotrigiano J. (2016). Regulation of retinoic acid inducible gene-I (RIG-I) activation by the histone deacetylase 6. *EBioMedicine* 9 195–206. 10.1016/j.ebiom.2016.06.015 27372014 PMC4972567

[B60] Liu H. M. Loo Y.-M. Horner S. M. Zornetzer G. A. Katze M. G. Gale M. (2012). The mitochondrial targeting chaperone 14-3-3ε regulates a RIG-I translocon that mediates membrane association and innate antiviral immunity. *Cell Host Microbe* 11 528–537. 10.1016/j.chom.2012.04.006 22607805 PMC3358705

[B61] Liu S. Cai X. Wu J. Cong Q. Chen X. Li T. (2015). Phosphorylation of innate immune adaptor proteins MAVS, STING, and TRIF induces IRF3 activation. *Science* 347:aaa2630. 10.1126/science.aaa2630 25636800

[B62] Liu S. Chen J. Cai X. Wu J. Chen X. Wu Y.-T. (2013). MAVS recruits multiple ubiquitin E3 ligases to activate antiviral signaling cascades. *Elife* 2:e00785. 10.7554/eLife.00785 23951545 PMC3743401

[B63] Liu X.-Y. Chen W. Wei B. Shan Y.-F. Wang C. (2011). IFN-induced TPR protein IFIT3 potentiates antiviral signaling by bridging MAVS and TBK1. *J. Immunol.* 187 2559–2568. 10.4049/jimmunol.1100963 21813773

[B64] Liu X.-Y. Wei B. Shi H.-X. Shan Y.-F. Wang C. (2010). Tom70 mediates activation of interferon regulatory factor 3 on mitochondria. *Cell Res.* 20 994–1011. 10.1038/cr.2010.103 20628368

[B65] Liuyu T. Yu K. Ye L. Zhang Z. Zhang M. Ren Y. (2019). Induction of OTUD4 by viral infection promotes antiviral responses through deubiquitinating and stabilizing MAVS. *Cell Res.* 29 67–79. 10.1038/s41422-018-0107-6 30410068 PMC6318273

[B66] Ma J. Ketkar H. Geng T. Lo E. Wang L. Xi J. (2018). Zika virus non-structural protein 4A blocks the RLR-MAVS Signaling. *Front. Microbiol.* 9:1350. 10.3389/fmicb.2018.01350 29988497 PMC6026624

[B67] Maharaj N. P. Wies E. Stoll A. Gack M. U. (2012). Conventional protein kinase C- (PKC-) and PKC- negatively regulate RIG-I antiviral signal transduction. *J. Virol.* 86 1358–1371. 10.1128/jvi.06543-11 22114345 PMC3264329

[B68] Mehta V. Trinkle-Mulcahy L. (2016). Recent advances in large-scale protein interactome mapping. *F1000Res.* 5:F1000FacultyRev–782. 10.12688/F1000RESEARCH.7629.1 27158474 PMC4856108

[B69] Meng J. Yao Z. He Y. Zhang R. Zhang Y. Yao X. (2017). ARRDC4 regulates enterovirus 71-induced innate immune response by promoting K63 polyubiquitination of MDA5 through TRIM65. *Cell Death Dis.* 8:e2866. 10.1038/cddis.2017.257 28594402 PMC5520913

[B70] Miyashita M. Oshiumi H. Matsumoto M. Seya T. (2011). DDX60, a DEXD/H Box helicase, is a novel antiviral factor promoting RIG-I-Like receptor-mediated signaling. *Mol. Cell. Biol.* 31 3802–3819. 10.1128/mcb.01368-10 21791617 PMC3165724

[B71] Mohanty A. Tiwari-Pandey R. Pandey N. R. (2019). Mitochondria: the indispensable players in innate immunity and guardians of the inflammatory response. *J. Cell Commun. Signal.* 13 303–318. 10.1007/s12079-019-00507-9 30719617 PMC6732146

[B72] Motwani M. Pesiridis S. Fitzgerald K. A. (2019). DNA sensing by the cGAS–STING pathway in health and disease. *Nat. Rev. Genet.* 20 657–674. 10.1038/s41576-019-0151-1 31358977

[B73] Mukherjee A. Morosky S. A. Delorme-Axford E. Dybdahl-Sissoko N. Oberste M. S. Wang T. (2011). The coxsackievirus B 3C protease cleaves MAVS and TRIF to attenuate host type I interferon and apoptotic signaling. *PLoS Pathog.* 7:e1001311. 10.1371/journal.ppat.1001311 21436888 PMC3059221

[B74] Narayan K. Waggoner L. Pham S. T. Hendricks G. L. Waggoner S. N. Conlon J. (2014). TRIM13 Is a negative regulator of MDA5-mediated type I interferon production. *J. Virol.* 88 10748–10757. 10.1128/jvi.02593-13 25008915 PMC4178852

[B75] Nistal-Villán E. Gack M. U. Martínez-Delgado G. Maharaj N. P. Inn K.-S. Yang H. (2010). Negative role of RIG-I serine 8 phosphorylation in the regulation of interferon-beta production. *J. Biol. Chem.* 285 20252–20261. 10.1074/jbc.M109.089912 20406818 PMC2888438

[B76] Odendall C. Dixit E. Stavru F. Bierne H. Franz K. M. Durbin A. F. (2014). Diverse intracellular pathogens activate type III interferon expression from peroxisomes. *Nat. Immunol.* 15 717–726. 10.1038/ni.2915 24952503 PMC4106986

[B77] Okamoto M. Kouwaki T. Fukushima Y. Oshiumi H. (2017). Regulation of RIG-I activation by K63-Linked polyubiquitination. *Front. Immunol.* 8:1942. 10.3389/fimmu.2017.01942 29354136 PMC5760545

[B78] Onoguchi K. Onomoto K. Takamatsu S. Jogi M. Takemura A. Morimoto S. (2010). Virus-infection or 5′ppp-RNA activates antiviral signal through redistribution of IPS-1 mediated by MFN1. *PLoS Pathog.* 6:e1001012. 10.1371/journal.ppat.1001012 20661427 PMC2908619

[B79] Oshiumi H. Kouwaki T. Seya T. (2016). Accessory factors of cytoplasmic viral RNA sensors required for antiviral innate immune response. *Front. Immunol.* 7:200. 10.3389/fimmu.2016.00200 27252702 PMC4879126

[B80] Oshiumi H. Matsumoto M. Hatakeyama S. Seya T. (2009). Riplet/RNF135, a RING finger protein, ubiquitinates RIG-I to promote interferon-beta induction during the early phase of viral infection. *J. Biol. Chem.* 284 807–817. 10.1074/jbc.M804259200 19017631

[B81] Pan Y. Li R. Meng J.-L. Mao H.-T. Zhang Y. Zhang J. (2014). Smurf2 negatively modulates RIG-I-dependent antiviral response by targeting VISA/MAVS for ubiquitination and degradation. *J. Immunol.* 192 4758–4764. 10.4049/jimmunol.1302632 24729608

[B82] Parisien J.-P. Lenoir J. J. Mandhana R. Rodriguez K. R. Qian K. Bruns A. M. (2018). RNA sensor LGP2 inhibits TRAF ubiquitin ligase to negatively regulate innate immune signaling. *EMBO Rep.* 19:e45176. 10.15252/embr.201745176 29661858 PMC5989757

[B83] Qi N. Shi Y. Zhang R. Zhu W. Yuan B. Li X. (2017). Multiple truncated isoforms of MAVS prevent its spontaneous aggregation in antiviral innate immune signalling. *Nat. Commun.* 8:15676. 10.1038/ncomms15676 28607490 PMC5474743

[B84] Qin Y. Xue B. Liu C. Wang X. Tian R. Xie Q. (2017). NLRX1 Mediates MAVS degradation to attenuate the hepatitis C virus-induced innate immune response through PCBP2. *J. Virol.* 91:e01264-17. 10.1128/jvi.01264-17 28956771 PMC5686720

[B85] Rebsamen M. Vazquez J. Tardivel A. Guarda G. Curran J. Tschopp J. (2011). NLRX1/NOD5 deficiency does not affect MAVS signalling. *Cell Death Differ.* 18:1387. 10.1038/cdd.2011.64 21617692 PMC3172102

[B86] Refolo G. Ciccosanti F. Di Rienzo M. Basulto Perdomo A. Romani M. Alonzi T. (2019). Negative regulation of mitochondrial antiviral signaling protein-mediated antiviral signaling by the mitochondrial protein LRPPRC during hepatitis C virus infection. *Hepatology* 69 34–50. 10.1002/hep.30149 30070380

[B87] Riedl W. Acharya D. Lee J.-H. Liu G. Serman T. Chiang C. (2019). Zika virus NS3 mimics a cellular 14-3-3-binding motif to antagonize RIG-I- and MDA5-mediated innate immunity. *Cell Host Microbe* 26 493.e6–503.e6. 10.1016/J.CHOM.2019.09.012 31600501 PMC6922055

[B88] Satoh T. Kato H. Kumagai Y. Yoneyama M. Sato S. Matsushita K. (2010). LGP2 is a positive regulator of RIG-I- and MDA5-mediated antiviral responses. *Proc. Natl. Acad. Sci. U.S.A.* 107 1512–1517. 10.1073/pnas.0912986107 20080593 PMC2824407

[B89] Seth R. B. Sun L. Ea C.-K. Chen Z. J. (2005). Identification and characterization of MAVS, a mitochondrial antiviral signaling protein that activates NF-kappaB and IRF 3. *Cell* 122 669–682. 10.1016/j.cell.2005.08.012 16125763

[B90] Sharma D. Kanneganti T. D. (2016). The cell biology of inflammasomes: mechanisms of inflammasome activation and regulation. *J. Cell Biol.* 213 617–629. 10.1083/jcb.201602089 27325789 PMC4915194

[B91] Shi Y. Yuan Zhu Zhang Li Hao (2017). Ube2D3 and Ube2N are essential for RIG-I-mediated MAVS aggregation in antiviral innate immunity. *Nat. Commun.* 8:15138. 10.1038/ncomms15138 28469175 PMC5418627

[B92] Soares F. Tattoli I. Wortzman M. E. Arnoult D. Philpott D. J. Girardin S. E. (2013). NLRX1 does not inhibit MAVS-dependent antiviral signalling. *Innate Immun.* 19 438–448. 10.1177/1753425912467383 23212541

[B93] Song T. Wei C. Zheng Z. Xu Y. Cheng X. Yuan Y. (2010). c-Abl tyrosine kinase interacts with MAVS and regulates innate immune response. *FEBS Lett.* 584 33–38. 10.1016/j.febslet.2009.11.025 19914245

[B94] Song N. Qi Q. Cao R. Qin B. Wang B. Wang Y. (2019). MAVS O-GlcNAcylation is essential for host antiviral immunity against lethal RNA viruses. *Cell Rep.* 28 2386.e5–2396.e5. 10.1016/j.celrep.2019.07.085 31461653

[B95] Soonthornvacharin S. Rodriguez-Frandsen A. Zhou Y. Galvez F. Huffmaster N. J. Tripathi S. (2017). Systems-based analysis of RIG-I-dependent signalling identifies KHSRP as an inhibitor of RIG-I receptor activation. *Nat. Microbiol.* 2:17022. 10.1038/nmicrobiol.2017.22 28248290 PMC5338947

[B96] Streicher F. Jouvenet N. (2019). Stimulation of innate immunity by host and viral RNAs. *Trends Immunol.* 40 1134–1148. 10.1016/j.it.2019.10.009 31735513

[B97] Subramanian N. Natarajan K. Clatworthy M. R. Wang Z. Germain R. N. (2013). The adaptor MAVS promotes NLRP3 mitochondrial localization and inflammasome activation. *Cell* 153 348–361. 10.1016/j.cell.2013.02.054 23582325 PMC3632354

[B98] Sun X. Sun L. Zhao Y. Li Y. Lin W. Chen D. (2016). MAVS maintains mitochondrial homeostasis via autophagy. *Cell Discov.* 2:16024. 10.1038/celldisc.2016.24 27551434 PMC4986202

[B99] Sun Z. Ren H. Liu Y. Teeling J. L. Gu J. (2011). Phosphorylation of RIG-I by casein kinase II inhibits its antiviral response. *J. Virol.* 85 1036–1047. 10.1128/jvi.01734-10 21068236 PMC3020001

[B100] Swanson K. V. Deng M. Ting J. P. Y. (2019). The NLRP3 inflammasome: molecular activation and regulation to therapeutics. *Nat. Rev. Immunol.* 19 477–489. 10.1038/s41577-019-0165-0 31036962 PMC7807242

[B101] Tait Wojno E. D. Hunter C. A. Stumhofer J. S. (2019). The immunobiology of the interleukin-12 family: room for discovery. *Immunity* 50 851–870. 10.1016/j.immuni.2019.03.011 30995503 PMC6472917

[B102] Takashima K. Oshiumi H. Takaki H. Matsumoto M. Seya T. (2015). RIOK3-mediated phosphorylation of MDA5 interferes with its assembly and attenuates the innate immune response. *Cell Rep.* 11 192–200. 10.1016/j.celrep.2015.03.027 25865883

[B103] Tal M. C. Iwasaki A. (2009). Autophagic control of RLR signaling. *Autophagy* 5 749–750. 10.4161/auto.5.5.8789 19571662 PMC3693554

[B104] Tan P. He L. Cui J. Qian C. Cao X. Lin M. (2017). Assembly of the WHIP-TRIM14-PPP6C mitochondrial complex promotes RIG-I-mediated antiviral signaling. *Mol. Cell* 68 293.e5–307.e5. 10.1016/j.molcel.2017.09.035 29053956

[B105] Tan X. Sun L. Chen J. Chen Z. J. (2018). Detection of microbial infections through innate immune sensing of nucleic acids. *Annu. Rev. Microbiol.* 72 447–478. 10.1146/annurev-micro-102215-095605 30200854

[B106] Tang E. D. Wang C.-Y. (2009). MAVS self-association mediates antiviral innate immune signaling. *J. Virol.* 83 3420–3428. 10.1128/jvi.02623 19193783 PMC2663242

[B107] Varga Z. T. Grant A. Manicassamy B. Palese P. (2012). Influenza virus protein PB1-F2 Inhibits the induction of Type I interferon by binding to MAVS and decreasing mitochondrial membrane potential. *J. Virol.* 86 8359–8366. 10.1128/jvi.01122-12 22674996 PMC3421771

[B108] Vazquez C. Horner S. M. (2015). MAVS coordination of antiviral innate immunity. *J. Virol.* 89 6974–6977. 10.1128/JVI.01918-14 25948741 PMC4473567

[B109] Venkataraman T. Valdes M. Elsby R. Kakuta S. Caceres G. Saijo S. (2007). Loss of DExD/H box RNA helicase LGP2 manifests disparate antiviral responses. *J. Immunol.* 178 6444–6455. 10.4049/jimmunol.178.10.6444 17475874

[B110] Vitour D. Dabo S. Ahmadi Pour M. Vilasco M. Vidalain P.-O. Jacob Y. (2009). Polo-like kinase 1 (PLK1) regulates interferon (IFN) induction by MAVS. *J. Biol. Chem.* 284 21797–21809. 10.1074/jbc.M109.018275 19546225 PMC2755906

[B111] Wagner S. A. Beli P. Weinert B. T. Schölz C. Kelstrup C. D. Young C. (2012). Proteomic analyses reveal divergent ubiquitylation site patterns in murine tissues. *Mol. Cell. Proteomics* 11 1578–1585. 10.1074/mcp.M112.017905 22790023 PMC3518112

[B112] Wang B. Xi X. Lei X. Zhang X. Cui S. Wang J. (2013). Enterovirus 71 protease 2A pro targets MAVS to inhibit anti-viral type I interferon responses. *PLoS Pathog.* 9:e1006243. 10.1371/journal.ppat.1003231 23555247 PMC3605153

[B113] Wang L. Zhao W. Zhang M. Wang P. Zhao K. Zhao X. (2013). USP4 positively regulates RIG-I-mediated antiviral response through deubiquitination and stabilization of RIG-I. *J. Virol.* 87 4507–4515. 10.1128/jvi.00031-13 23388719 PMC3624380

[B114] Wang P. Yang L. Cheng G. Yang G. Xu Z. You F. (2013). UBXN1 interferes with Rig-I-like receptor-mediated antiviral immune response by targeting MAVS. *Cell Rep.* 3 1057–1070. 10.1016/j.celrep.2013.02.027 23545497 PMC3707122

[B115] Wang W. Jiang M. Liu S. Zhang S. Liu W. Ma Y. (2016). RNF122 suppresses antiviral type I interferon production by targeting RIG-I CARDs to mediate RIG-I degradation. *Proc. Natl. Acad. Sci. U.S.A.* 113 9581–9586. 10.1073/pnas.1604277113 27506794 PMC5003265

[B116] Wang Y. Tong X. Ye X. (2012). Ndfip1 negatively regulates RIG-I-dependent immune signaling by enhancing E3 ligase Smurf1-mediated MAVS degradation. *J. Immunol.* 189 5304–5313. 10.4049/jimmunol.1201445 23087404

[B117] West A. P. Brodsky I. E. Rahner C. Woo D. K. Erdjument-Bromage H. Tempst P. (2011). TLR signalling augments macrophage bactericidal activity through mitochondrial ROS. *Nature* 472 476–480. 10.1038/nature09973 21525932 PMC3460538

[B118] Wi S. M. Moon G. Kim J. Kim S. T. Shim J. H. Chun E. (2014). TAK1-ECSIT-TRAF6 complex plays a key role in the TLR4 signal to activate NF-κB. *J. Biol. Chem.* 289 35205–35214. 10.1074/jbc.M114.597187 25371197 PMC4271209

[B119] Wies E. Wang M. K. Maharaj N. P. Chen K. Zhou S. Finberg R. W. (2013). Dephosphorylation of the RNA sensors RIG-I and MDA5 by the phosphatase PP1 is essential for innate immune signaling. *Immunity* 38 437–449. 10.1016/j.immuni.2012.11.018 23499489 PMC3616631

[B120] Willemsen J. Wicht O. Wolanski J. C. Baur N. Bastian S. Haas D. A. (2017). Phosphorylation-dependent feedback inhibition of RIG-I by dapk1 identified by kinome-wide siRNA Screening. *Mol. Cell* 65 403.e8–415.e8. 10.1016/j.molcel.2016.12.021 28132841

[B121] Wu J. Sun L. Chen X. Du F. Shi H. Chen C. (2013). Cyclic GMP-AMP is an endogenous second messenger in innate immune signaling by cytosolic DNA. *Science* 339 826–830. 10.1126/science.1229963 23258412 PMC3855410

[B122] Xian H. Yang S. Jin S. Zhang Y. Cui J. (2019). LRRC59 modulates type I interferon signaling by restraining the SQSTM1/p62-mediated autophagic degradation of pattern recognition receptor DDX58/RIG-I. *Autophagy* 16 408–418. 10.1080/15548627.2019.1615303 31068071 PMC6999607

[B123] Xiang W. Zhang Q. Lin X. Wu S. Zhou Y. Meng F. (2016). PPM1A silences cytosolic RNA sensing and antiviral defense through direct dephosphorylation of MAVS and TBK1. *Sci. Adv.* 2:e1501889. 10.1126/sciadv.1501889 27419230 PMC4942338

[B124] Xing J. Zhang A. Minze L. J. Li X. C. Zhang Z. (2018). TRIM29 Negatively Regulates the Type I IFN Production in Response to RNA Virus. *J. Immunol.* 201 183–192. 10.4049/jimmunol.1701569 29769269 PMC6092021

[B125] Xu L. Xiao N. Liu F. Ren H. Gu J. (2009). Inhibition of RIG-I and MDA5-dependent antiviral response by gC1qR at mitochondria. *Proc. Natl. Acad. Sci. U.S.A.* 106 1530–1535. 10.1073/pnas.0811029106 19164550 PMC2635802

[B126] Xue B. Li H. Guo M. Wang J. Xu Y. Zou X. (2018). TRIM21 Promotes Innate Immune Response to RNA Viral Infection through Lys27-Linked Polyubiquitination of MAVS. *J. Virol.* 92:e00321-18. 10.1128/jvi.00321-18 29743353 PMC6026736

[B127] Yang Y. Liang Y. Qu L. Chen Z. Yi M. Li K. (2007). Disruption of innate immunity due to mitochondrial targeting of a picornaviral protease precursor. *Proc. Natl. Acad. Sci. U.S.A.* 104 7253–7258. 10.1073/pnas.0611506104 17438296 PMC1855380

[B128] Yasukawa K. Oshiumi H. Takeda M. Ishihara N. Yanagi Y. Seya T. (2009). Mitofusin 2 inhibits mitochondrial antiviral signaling. *Sci. Signal.* 2:ra47. 10.1126/scisignal.2000287 19690333

[B129] Yates J. R. (2019). Recent technical advances in proteomics. *F1000Res.* 8:F1000FacultyRev–351. 10.12688/f1000research.16987.1 30997033 PMC6441878

[B130] Yoneyama M. Kikuchi M. Matsumoto K. Imaizumi T. Miyagishi M. Taira K. (2005). Shared and unique functions of the DExD/H-box helicases RIG-I. MDA5, and LGP2 in Antiviral Innate Immunity. *J. Immunol.* 175 2851–2858. 10.4049/jimmunol.175.5.2851 16116171

[B131] Yoo Y.-S. Park Y.-Y. Kim J.-H. Cho H. Kim S.-H. Lee H.-S. (2015). The mitochondrial ubiquitin ligase MARCH5 resolves MAVS aggregates during antiviral signalling. *Nat. Commun.* 6:7910. 10.1038/ncomms8910 26246171 PMC4918326

[B132] Yoshinaka T. Kosako H. Yoshizumi T. Furukawa R. Hirano Y. Kuge O. (2019). Structural basis of mitochondrial scaffolds by prohibitin complexes: insight into a role of the coiled-coil region. *iScience* 19 1065–1078. 10.1016/j.isci.2019.08.056 31522117 PMC6745515

[B133] Yoshizumi T. Imamura H. Taku T. Kuroki T. Kawaguchi A. Ishikawa K. (2017). RLR-mediated antiviral innate immunity requires oxidative phosphorylation activity. *Sci. Rep.* 7:5379 28710430 10.1038/s41598-017-05808-w PMC5511143

[B134] You F. Sun H. Zhou X. Sun W. Liang S. Zhai Z. (2009). PCBP2 mediates degradation of the adaptor MAVS via the HECT ubiquitin ligase AIP4. *Nat. Immunol.* 10 1300–1308. 10.1038/ni.1815 19881509

[B135] Zevini A. Olagnier D. Hiscott J. (2017). Crosstalk between Cytoplasmic RIG-I and STING Sensing Pathways. *Trends Immunol.* 38 194–205. 10.1016/j.it.2016.12.004 28073693 PMC5329138

[B136] Zhang W. Wang G. Xu Z.-G. Tu H. Hu F. Dai J. (2019). lactate is a natural suppressor of RLR signaling by targeting MAVS. *Cell* 178 176.e15–189.e15. 10.1016/j.cell.2019.05.003 31155231 PMC6625351

[B137] Zhao C. Jia M. Song H. Yu Z. Wang W. Li Q. (2017). The E3 ubiquitin ligase TRIM40 attenuates antiviral immune responses by targeting MDA5 and RIG-I. *Cell Rep.* 21 1613–1623. 10.1016/j.celrep.2017.10.020 29117565

[B138] Zhao K. Zhang Q. Li X. Zhao D. Liu Y. Shen Q. (2016). Cytoplasmic STAT4 promotes antiviral Type I IFN production by blocking CHIP-mediated degradation of RIG-I. *J. Immunol.* 196 1209–1217. 10.4049/jimmunol.1501224 26695369

[B139] Zhao Y. Sun X. Nie X. Sun L. Tang T.-S. Chen D. (2012). COX5B regulates MAVS-mediated antiviral signaling through interaction with ATG5 and repressing ROS production. *PLoS Pathog.* 8:e1003086. 10.1371/journal.ppat.1003086 23308066 PMC3534373

[B140] Zhong B. Zhang Y. Tan B. Liu T.-T. Wang Y.-Y. Shu H.-B. (2010). The E3 ubiquitin ligase RNF5 targets virus-induced signaling adaptor for ubiquitination and degradation. *J. Immunol.* 184 6249–6255. 10.4049/jimmunol.0903748 20483786

[B141] Zhou P. Ding X. Wan X. Liu L. Yuan X. Zhang W. (2018). MLL5 suppresses antiviral innate immune response by facilitating STUB1-mediated RIG-I degradation. *Nat. Commun.* 9:1243. 10.1038/s41467-018-03563-8 29593341 PMC5871759

[B142] Zhou Z. Jia X. Xue Q. Dou Z. Ma Y. Zhao Z. (2014). TRIM14 is a mitochondrial adaptor that facilitates retinoic acid-inducible gene-I-like receptor-mediated innate immune response. *Proc. Natl. Acad. Sci. U.S.A.* 111 E245–E254. 10.1073/pnas.1316941111 24379373 PMC3896185

